# Association of social support and religiosity with survival among women with breast cancer in a low-income population in the Southeastern United States

**DOI:** 10.1186/s12889-025-21908-z

**Published:** 2025-02-20

**Authors:** Ronald Fisa, Kabisa Mwala, Douglas DeMoulin, Violet Kayamba, Martha Shrubsole, Xiao-Ou Shu, Isaac Fwemba, Wilbroad Mutale, Loren Lipworth

**Affiliations:** 1 Department of Epidemiology and Biostatistics, School of Public Health, University of Zambia, Lusaka, Zambia; 2 Surgical and Gynaecologic Oncology Department, Cancer Diseases Hospital, Lusaka, Zambia; 3 Division of Epidemiology, Department of Medicine, Vanderbilt University Medical Center, Nashville, TN USA; 4 Department of Internal Medicine, School of Medicine, University of Zambia, Lusaka, Zambia; 5 Department of Health Policy and Management, School of Public Health, University of Zambia, Lusaka, Zambia

**Keywords:** Breast cancer, Survival, Psychosocial factors, Social support, Religiosity

## Abstract

**Background:**

Large social networks have been associated with better overall survival after a breast cancer diagnosis in some but not all study populations. This study evaluated associations of social support and religiosity/spirituality with survival among Black and White women with breast cancer of largely low socioeconomic status in the United States (US).

**Methods:**

The study used data from the prospective Southern Community Cohort Study, which enrolled approximately 86,000 adults in the southeastern US during 2002–2009. A total of 1,347 Black and White women with incident breast cancer were identified in the cohort and followed through December 2020 for mortality via linkage with the National Death Index. Exposures of interest were social support and religiosity obtained via baseline questionnaire, including number of close friends/relatives who can provide instrumental and emotional support, and frequency of attendance at religious services. Multivariable Cox regression models were used to estimate hazard ratios (HR) and 95% confidence intervals (CI) for all-cause mortality in association with social support and religiosity. The models were tested for proportional hazards assumption using Schoenfeld residuals.

**Results:**

Among the 1,347 women with breast cancer, 365 (27.1%) died during follow up. The participants were followed up for 17 years with a median follow-up time of 5 years. In all-cause mortality analyses, women who reported having 2 + relatives/friends for emotional support had a 20% reduced hazard of death compared to women with *≤* 1 relative/friend (HR = 0.80, 95% CI: 0.67–0.96) after adjusting for age at breast cancer diagnosis, race, time from cohort enrollment to diagnosis, income, education, marital status, insurance, and tumor hormone receptor status. Similarly, women reporting having 2 + people able to provide instrumental support (render assistance in an emergency or lend money) had a 25% (HR = 0.75, 95% CI: 0.59–0.95) reduced hazard of death compared to those who had *≤* 1. Frequent attendance at religious services/meetings was associated with reduced hazard of death compared to those who did not attend (HR = 0.60, CI: 0.41–0.89); addition of cancer stage in the models attenuated this association.

**Conclusions:**

A large social support network and regular attendance at faith-based services were associated with better survival among women with breast cancer. This calls for incorporating appropriate interventions to cancer care such as social support groups to improve survival.

**Supplementary Information:**

The online version contains supplementary material available at 10.1186/s12889-025-21908-z.

## Introduction

Breast cancer (BC) is the leading cancer among women globally, affecting more than one in ten women [1, 2]. In the United States (US), BC is the most commonly diagnosed cancer in women after skin cancers [2] and the American Cancer Society estimated that in 2023 there would be 297,790 new cases of invasive BC. In the past two decades, BC deaths have substantially reduced due to the uptake of early screening for BC as well as improved cancer diagnostic and treatment advancements [3, 4]. Even with improved survival, women with BC may experience adverse effects from treatments such as chemotherapy and radiotherapy and many women report reduced quality of life (QoL) and suffer from depression/anxiety [5].

Several factors that affect the survival of women with BC, such as age, cancer stage, metastatic state, tumor size and hormone receptor status are well documented [6–8]. Conversely, findings from empirical research are equivocal on the effect of social support on survival of women with BC. For example, studies conducted in developed countries have shown evidence that greater contact with friends and family reduced the risk of dying [8, 9], while other studies have reported contradicting results particularly with respect to faith or religiosity [10]. One Study that examined the psychological state of cancer survivors found a presence of mood changes in the women survivors’ financial burden as well as physical changes due to the treatment they went through [11]. A need for psychosocial care and support has been documented among underserved BC survivors [11] as they may be afraid of the recurrence of BC and the effect of the treatment on the bodies of these survivors.

Compared to White women, Black women in the US are more likely to be diagnosed with BC at earlier ages and with aggressive subtypes, and Black women with BC have lower survival rates than White women [1, 12–14]. Further, a study by Assari [15] found that Black women with BC had higher depressive symptoms compared to their White counterparts, but high depressive symptoms increased the short-term risk of mortality for White but not Black older adults. Research has also indicated that lack of private insurance and higher prevalence of comorbidities contribute to the lower survival in Black Americans [16, 17].

In this study we examined the association between social support and religiosity with all-cause and breast cancer specific mortality among White and Black women with BC of generally low socioeconomic status (SES). We hypothesized that social support and religiosity positively influence survival of women with BC.

## Methods

### Setting and study population

Women diagnosed with BC were drawn from the Southern Community Cohort Study (SCCS), a prospective cohort study designed to investigate health disparities in a low-income and underserved population. Details of the study design and implementation of the SCCS have been reported elsewhere [18]. In brief, the SCCS enrolled approximately 86,000 participants during 2002 and 2009, aged 40 to 79 years, 2/3 of whom reported their race as Black or African American. Participants were recruited predominantly through Community Health Centers in 12 states in the southeastern United States (Alabama, Arkansas, Florida, Georgia, Kentucky, Louisiana, Mississippi, North Carolina, South Carolina, Tennessee, Virginia, and West Virginia). This analysis was restricted to all 1,347 women who were diagnosed with incident BC during cohort follow up to December 31, 2018, via linkage of the cohort with state cancer registries.

All participants provided written informed consent. Protocols were approved by the Institutional Review Boards of Vanderbilt University Medical Center and Meharry Medical College.

### Exposure variables

At the time of the main cohort enrollment, all SCCS participants completed detailed questionnaires ascertaining demographic, socioeconomic, behavioral, psychosocial and health history information [18]. The survey questions that were used were obtained/modified or developed from various sources by the research team and can be obtained on the SCCS website [19]. Specifically, for this study we used the women’s questionnaire (supplementary file 1) which consisted of questions on social, lifestyle and religiosity variables in addition to clinical factors. Participants were asked a question about emotional support “*How many close friends or relatives would help you with your emotional problems or feelings if you need it?*”. Participants were also asked about instrumental support “*How many people could you ask for help in an emergency or with lending you money*?”. For both questions, the response was categorized into 0 to 1 and 2 or more people. For the purposes of this study, women who had 2 or more relatives/friends were regarded to have a large social network. The measures used for religiosity [20] and social support in this study were adopted from scales that have been validated elsewhere. In addition, these scales were also assessed for reliability in the SCCS [18].

With respect to religiosity and spirituality, participants were asked three questions at the time of cohort enrollment. The first question was “*How spiritual or religious do you consider yourself to be?*” The responses to this question were 0 = “not at all/slightly”, 1 = “fairly”, and 2 = “very religious”. Participants were also asked “*How much is religion*,* faith*,* or God a source of strength and comfort to you?*”. Responses were categorized as 0 = “not very much/somewhat”, 1 = “quite a bit” and 2 = “a great deal”. Lastly, participants were asked *“How often do you attend religious or faith services during the year?”* Responses were categorized as 0 = “never”, 1 = “on holidays only”, 2 = “once per week” and 3 = “greater than once per week”. The frequency of church attendance in this study referred to attending religious or faith services greater than 3 times per week.

### Outcome ascertainment

The primary outcome was all-cause mortality after BC diagnosis. The secondary outcome was BC-specific mortality (BC as the cause of death). Information on dates and causes of death was ascertained via linkage of the SCCS cohort with the National Death Index (NDI) through December 31, 2020. Person-months of follow-up began on the participant’s date of diagnosis with BC and ended on the date of death or December 31, 2020, whichever occurred first.

### Statistical analysis

In descriptive analyses, we used frequencies and percentages to describe the distribution of categorical study variables. Continuous variables were categorized as they were not normally distributed. In establishing if there were any differences in the proportions of women who had ≥ 2 or ≤ 1 relative for social support and other covariates, a chi-squared test was used. For the main exposure variables of social support and religiosity, we compared the survival of two groups of women with BC, for example, those with *≤*1 and those with ≥ 2 supporting individuals. The Log-rank test was used to draw the survival curves and assess if the difference in survival was statistically significant, given the level of social support or religiosity. Multicollinearity was assessed using the Variance Inflation Factor (VIF) on all the social and religiosity variables, these included marital status, help in emergencies, social support and others. Multivariable Cox regression models were used to estimate hazard ratios and 95% confidence intervals for all-cause and BC-specific mortality associated with social support and religiosity. The proportional hazards assumption was checked using Schoenfield residuals. In Model 1, we evaluated the effect of social support or religiosity on survival of women living with BC controlling for age at BC diagnosis, race, and time from cohort enrollment to diagnosis. In Model 2 we controlled for the variables in Model 1 in addition to income, education, marital status, insurance. In Model 3, we further adjusted for variables in Model 2 and added clinical variables such as HER2, tumor estrogen receptor (ER) status and tumor progesterone receptor (PR) status. In model 4 we adjusted for all the variables in model 3 in addition of treatment, cancer stage and co-morbidities. The models were adjusted for confounding as social support of women with BC may differ depending on treatment received, hormone status, co-morbidities and other clinical variables on an individual as well as to improve the accuracy of the associations observed [21]. In order to accurately estimate the effect of social support on survival, we controlled for clinical variables such as treatment, cancer stage as some patients may have a large network of friends tend to be diagnosed with early-stages of BC [22]. In the BC-specific mortality analysis, all women who died of causes other than BC, such as heart failure or hypertension, were treated as censored individuals at the time of death. In the BC specific analysis, we adjusted for age at diagnosis, race, and time from cohort enrollment to diagnosis, income, education, marital status, insurance, cancer stage, tumor estrogen receptor (ER) status and tumor progesterone receptor (PR) status. We then used the Schoenfeld residuals to test for the proportional hazards assumption for the goodness-of-fit of the Models fitted. The best fit model was also assessed using the likelihood ratio test comparing the full model and the null models. We performed analyses in R version 4.3.1 “Beagle Scouts”, The R project, Vienna, Austria. All statistical tests used were 2-sided probability, and all P-values less than 0.05 were considered as statistically significant.

## Results

### Baseline characteristics of the participants

Of the 1,347 women with BC included in our analysis, 365 died and 982 were alive at the end of follow up. The participants were followed up for approximately 205 months (17 years) and the median follow-up time was 60 months (5 years). The majority (35.2%) of the women with BC were in the age range 50–59 years at BC diagnosis, and 69.2% of them were Black women. Table 1 presents the baseline characteristics of the study population overall and by vital status. Overall, 54% of women had annual household income less than or equal to $15,000. Among the 365 women who died, the proportion with household income less than or equal to $15,000 increased to 66.8%.

**Table 1:** Baseline characteristics of women with BC by all-cause mortality

	All participants		Alive	Dead	
Variable	*N* = 1, 347	(%)	*N* = 982 (%)	*N* = 365 (%)	*P*-value
**Social support**: 0 to 1 2 or more	236 1111	(17.5) (82.5)	159 (16.2) 823 (83.8)	77 (21.1) 288 (78.9)	0.043^*^
**Help in Emergency**: 0 to 1 2 or more	342 982	(25.8) (74.2)	234 (24.3) 730 (75.7)	108 (30.0) 252 (70.0)	0.041^*^
**Spiritual**: Very Slightly/Fairly Not at all / Slightly	835 388 106	(62.8) (29.2) (8.0)	588 (60.7) 302 (31.2) 79 (8.1)	247 (68.6) 86 (23.9) 27 (7.5)	0.023^*^
**Faith service**: Never Holidays only Once per week > once per week	137 43 377 405 385	(10.2) (3.2) (28.0) (30.1) (28.5)	91 (9.3) 32 (3.2) 279 (28.4) 299 (30.4) 281 (28.6)	46 (12.6) 11 (3.0) 98 (26.8) 106 (29.0) 104 (28.5)	0.498
**Comfort in faith**: Somewhat A bit A great deal	103 210 1021	(7.7) (15.7) (76.5)	83 (8.5) 147 (15.1) 742 (76.4)	20 (5.5) 63 (17.4) 279 (77.1)	0.136
**Race**: White Black	415 932	(30.8) (69.2)	311 (31.7) 671 (68.3)	104 (28.5) 261 (71.5)	0.291
**Age at diagnosis**: 40–49 50–59 60–69 75+	140 474 463 270	(10.4) (35.2) (34.4) (20.0)	92 (9.4) 357 (36.4) 354 (36.0) 179 (18.2)	48 (13.4) 117 (33.1) 109 (30.9) 79 (22.4)	0.003^*^
**BMI**: Less than 25 25 to < 30 30 to < 35 35 to < 40 Over 40	212 338 341 220 218	(16.0) (25.4) (25.7) (16.6) (16.4)	145 (14.9) 254 (26.1) 251 (25.8) 164 (16.8) 159 (16.3)	67 (18.8) 84 (23.6) 90 (25.3) 56 (15.7) 59 (16.6)	0.503
**Marital Status**: Married Separated/Divorced Widowed Single	434 443 236 221	(32.5) (33.2) (17.7) (16.6)	346 (35.5) 321 (33.0) 152 (15.6) 155 (15.9)	88 (24.4) 122 (33.9) 84 (23.3) 66 (18.3)	< 0.001^*^
**Education**: LT HS HS/Training Some College College grad+	333 530 285 198	(24.7) (39.4) (21.2) (14.7)	222 (22.6) 382 (39.9) 208 (21.2) 169 (17.2)	111 (30.4) 148 (40.5) 77 (21.1) 29 (7.9)	<0.001^*^
**Household Income**: Less than or equal to $15,000 Greater than $15,000	717 607	(54.2) (45.8)	482 (49.6) 490 (50.4)	235 (66.8) 117 (33.2)	< 0.001^*^
**Insurance coverage**: No Yes	423 910	(31.7) (68.3)	318 (32.4) 654 (67.6)	105 (21.1) 256 (70.9)	0.230
**Smoking status**: Never smoked. Former Current	375 316 649	(28.0) (23.6) (48.4)	260 (26.6) 227 (23.3) 489 (50.1)	115 (31.6) 89 (24.4) 160 (44.0)	0.103
**Cancer stage**: I II III/IV Unknown	408 356 221 362	(30.3) (26.4) (16.4) (26.9)	349 (35.3) 256 (26.1) 79 (8.4) 298 (30.3)	59 (16.2) 100 (27.4) 142 (38.9) 64 (17.5)	< 0.001^*^
**Clinical result**: ER + and/or PR+/HER2- HER2+/ER + and/or PR+ ER-/PR-/HER2+ ER-/PR-/HER2- Any missing	648 112 71 198 318	(48.0) (8.3) (5.2) (14.9) (23.6)	481 (49.0) 75 (7.6) 44 (4.5) 119 (12.1) 263 (26.8)	167 (45.8) 37 (10.1) 27 (7.4) 79 (21.6) 55 (15.1)	0.807
**Surgery**: No Yes Unknown	118 1143 86	(8.7) (84.8) (6.4)	52 (5.3) 869 (88.5) 61 (6.2)	66 (18.1) 274 (75.1) 25 (6.8)	< 0.001^*^
**Radiation**: No Yes Unknown	619 427 301	(46.0) (31.7) (22.3)	428 (43.6) 323 (32.9) 231 (23.5)	191 (52.3) 104 (28.5) 70 (19.2)	0.016^*^
**Chemotherapy**: No Yes Unknown	602 546 199	(44.7) (40.5) (14.8)	457 (46.5) 381 (38.8) 144 (14.7)	145 (39.7) 165 (45.2) 55 (15.1)	0.064
**Hormonal therapy**: No Yes Unknown	670 454 223	(49.7) (33.7) (16.6)	467 (47.6) 350 (35.6) 165 (16.8)	203 (55.6) 104 (28.5) 58 (15.9)	0.022^*^

*Variables statistically significant at α = 0.05 using a chi-squared test

As shown in Table 1, of the 1,347 women with BC overall, 236 (17.5%) had *≤* 1 friends/relatives offering them social support and 1111 (82.5%) had 2 or more friends/relatives to offer them social support. In terms of help in emergency, 342 (25.8%) had *≤* 1 person to help in an emergency while 982 (74.2%) had ≥ 2 help in emergency. Stratifying by the outcome (death) we observed that out of all the women who died, higher proportion of these had 2 or more helpers (70%) compared to those who only had 1 or no helper (30%). With respect to religiosity, overall, most of the participants indicated that they were very spiritual (62.8%) while 29.2% were slightly/fairly spiritual and 8% were not spiritual at all. Overall, 405 (30.1%) participants attended religious meetings/faith services once per week, while 385 (28.9%) attended more than once per week and 137 (10.2%) participants never attended.

Table 1 also shows that compared to women who were alive, those who died had an older age at diagnosis and were more likely to have higher education and low income. Specifically, age at diagnosis, out of the 365 women who died at the time of analysis, majority (33.1%) were aged 50 to 59 years of age and 30.9% were between 60 and 69 years old. In addition, the proportion of women who were married, and those with stage I cancer was lower among those who died compared to those who were alive.

### Comparing survival probability of groups using log-rank test

**Fig. 1:**
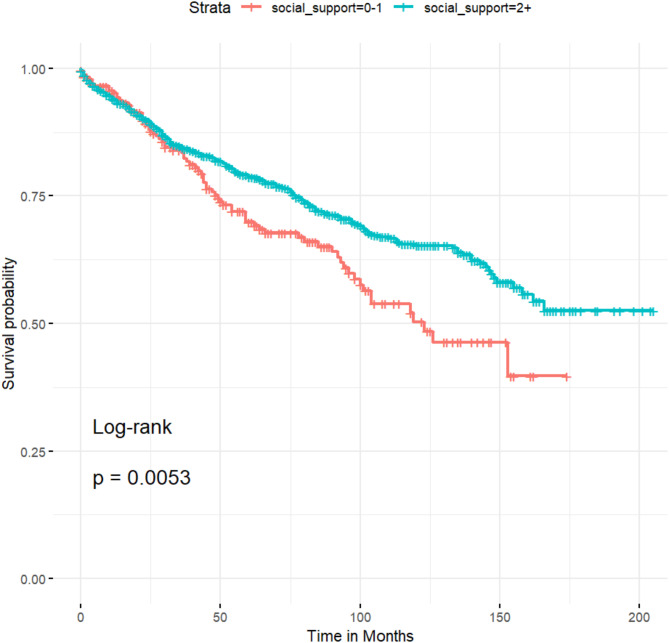
Survival of women with BC with social support of 0 to 1 compared to those with 2 + relatives/friends

The survival of women with/without social support was compared using the log-rank test (Fig. 1). In this comparison, we found that women with greater emotional support (≥ 2 relatives/friends) had better survival over time compared to those with fewer relatives or friends. This survival difference was statistically significant with p-value = 0.005. The survival curves are illustrated in Fig. 1 below.

We also compared survival of women with BC across the spirituality variable (results not shown). Using the Log-rank test we found no significant difference in the survival curves of women with BC who reported being not at all spiritual, slightly spiritual and very spiritual, p-value > 0.05.

### Social support and spirituality with other covariates

Table 2 presents the distribution of individual characteristics of study participants by level of social support. Out of the 236 women who had *≤* 1 friends/relatives to provide social support, 164 (69.5%) were Black women. For spirituality, out of 236 women who received less than or equal to 1 relatives/friends social support, 120/236 (51.1%) were not at all spiritual, while 77/236 (32.8%) were fairly spiritual and 38/236 (16.1%) women reported to be very spiritual. Out of the 1,111 women who received greater than 2 relatives/friends in terms of social support, 715/1111 (65.4%) were not at all spiritual, 311/1111 (28.4%) were fairly spiritual and 68/1111 (6.2%) reported to be very spiritual. The proportion of women who were very spiritual was lower in women who had ≥ 2 social support (6.2%) compared to those who had less than or equal to 1 (16.1%). The proportion of women with BC who attended religious/faith services more than once per week, had 2 or more individuals to help in emergency, got a great deal of comfort in faith and had income over $15,000 was greater in the women who had over ≥ 2 individuals to provide social support compared to those with *≤*1.

**Table 2:** Association of Social support and other covariates

	Social support (number of people)				
	0 to 1 *N* = 236		2 or more *N* = 1,111		*P*-value
Variable	*N*	%	*N*	%	
**Help in Emergency**: 0 to 1 2 or more	154 80	(65.8) (34.2)	188 902	(17.2) (82.8)	< 0.001^*^
**Spirituality**: Not at all / slightly Fairly Very Spiritual	120 77 38	(51.1) (32.8) (16.1)	715 311 68	(65.4) (28.4) (6.2)	< 0.001^*^
**Faith service**: Never Holidays only Once per week > once per week	44 82 61 49	(18.6) (34.7) (25.8) (20.8)	93 338 344 336	(8.4) (30.4) (30.9) (30.2)	< 0.001^*^
**Comfort in faith**: Somewhat A bit A great deal	23 50 162	(9.8) (21.3) (68.9)	80 160 859	(7.3) (14.5) (78.2)	< 0.001^*^
**Race**: White Black	72 164	(30.5) (69.5)	343 768	(30.9) (69.1)	0.974
**Marital Status**: Married Separated/Divorced Widowed Single	71 78 37 48	(30.3) (33.3) (15.8) (20.5)	363 365 199 173	(33.0) (33.2) (18.1) (15.7)	0.301
**Age at diagnosis**: 40–49 50–59 60–69 75+	30 83 81 42	(12.7) (35.2) (34.3) (17.8)	110 391 382 228	(9.9) (35.2) (34.4) (20.5)	0.532
Household Income: $15,000 >$15,000	157 75	(67.7) (32.3)	560 532	(51.3) (48.7)	< 0.001^*^
**Education**: LT HS HS/Training Some College College grad+	83 92 42 19	(35.2) (38.9) (17.8) (8.1)	250 438 243 179	(22.5) (39.4) (21.9) (16.1)	< 0.001^*^
**BMI**:: Less than 25 25 to < 30 30 to < 35 35 to < 40 Over 40	35 54 69 31 42	(15.2) (23.4) (29.9) (13.4) (18.1)	177 284 272 189 176	(16.2) (25.8) (24.8) (17.2) (16.0)	0.319
**Insurance**: No Yes	84 152	(35.6) (64.4)	339 772	(30.5) (69.5)	0.147
**Smoking status**: Never smoked. Former Current	80 57 98	(34.0) (24.3) (41.7)	295 259 551	(26.7) (23.4) (48.9)	0.039^*^
**Clinical result**: ER + and/orPR+/HER2- HER ≥ 2/ER + and/or PR+ ER-/PR-/HER ≥ 2 ER-/PR-/HER2- Any missing	113 17 15 38 53	(47.9) (7.2) (6.4) (16.1) (22.4)	535 95 56 160 265	(48.1) (8.6) (5.0) (14.4) (23.9)	0.807
**Cancer stage**: I II III/IV Unstaged	60 65 43 68	(25.4) (27.5) (18.2) (28.8)	348 291 178 294	(31.3) (26.2) (16.0) (26.5)	0.461
**Surgery**: No Yes Unknown	18 199 19	(7.6) (84.3) (8.1)	100 944 67	(9.0) (85.0) (6.0)	0.433
**Radiation**: No Yes Unknown	97 84 55	(41.1) (35.6) (23.3)	522 343 246	(47.0) (30.9) (22.1)	0.229
**Chemotherapy**: No Yes Unknown	98 103 35	(44.5) (43.6) (14.8)	504 443 164	(45.4) (39.9) (14.7)	0.516
**Hormonal therapy**: No Yes Unknown	122 72 42	(51.7) (30.5) (17.8)	548 382 181	(49.3) (34.4) (16.3)	0.508
**Hypertension**: No Yes	77 159	(32.6) (67.4)	402 709	(36.2) (63.8)	0.336

*Variables statistically significant at α = 0.05 using a chi-squared test

In a cross-tabulation analysis using chi-square test, we found that spirituality, faith service large, comfort in faith, income, education and smoking status of the women were significantly associated with social support (Table 2).

### All-cause mortality analysis

Table 3 presents estimates derived from multivariable models of association between measures of social support and religiosity in relation to all-cause mortality. The proportional hazards assumption was held in Model 3 and there was no evidence of multicollinearity between the social support and religiosity variables (all VIF values were < 1.05). Using the likelihood ratio test, model 3 was found to be the best fit model. Results from this model suggested that, compared to having ≤ 1 friends or relatives, having 2 or more friends or relatives for emotional support was associated with a significant 20% reduced hazard of all-cause mortality (Model 3) after adjustment for age at BC diagnosis, race, time from enrollment to diagnosis, income, education, marital status, insurance, HER2, tumor ER and PR status (HR 0.80; 95% CI 0.67–0.96). Similarly, women having 2 or more people to provide instrumental support had a 25% reduced hazard from all-cause mortality compared to those with 0–1 people (HR = 0.75, 95% CI: 0.59–0.95). For both social support variables, after further adjustment in model 4 (results not shown) for cancer stage at diagnosis, the association was attenuated and no longer statistically significant (HR = 0.86, CI: 0.70–1.05 and HR = 0.87, CI: 0.67–1.12, respectively). Women who attended religious services on holidays (HR = 0.61, 95% CI: 0.43–0.88), once per week (HR = 0.65, 95% CI: 0.45–0.95) and more than once per week (HR = 0.60, 95% CI: 0.41–0.89) had significant 35–40% reduced hazards from all-cause mortality compared to those who never attended religious services at all, adjusting for age at BC diagnosis, race, time from cohort enrollment to diagnosis, income, education, marital status, insurance, HER2, tumor ER and PR status However, addition of treatment and cancer stage in the fourth model attenuated this association. Neither spirituality nor drawing comfort from faith was significantly associated with all-cause mortality. Further, Model 4 violated the proportional hazards assumption as the predictor variables were varying over time.

**Table 3:** Association between social support and religiosity characteristics and all-cause mortality among participants

Psychosocial Variables	All Women with Breast cancer			
	Deaths/1347	HR^1^ (95% CI)	HR^2^ (95% CI)	HR^3^ (95% CI)
**Emotional support: How many close friends or relatives would help you with your emotional problems or feelings if you need it?**				
0 to 1	77/236	Ref	Ref	Ref
2 or more	288/1111	**0.76** **0.64–0.91**	**0.82** **0.69–0.98**	**0.80** **0.67–0.96**
**Instrumental support: How many people could you ask for help in an emergency or with lending you money?**				
None to 1	108/342	Ref.	Ref.	Ref
$\:\ge\:$ 2	252/982	**0.69** **0.55–0.86**	**0.77** **0.61–0.97**	**0.75** **0.59–0.95**
**Perceived spirituality: How spiritual or religious do you consider yourself to be?**				
Not at all/slightly	27/106	Ref	Ref	Ref
Fairly	86/388	0.99 0.75–1.33	1.05 0.79–1.41	1.07 0.80–1.43
Very	252/853	1.19 0.94–1.51	1.17 0.92–1.49	1.16 0.91–1.47
***Perceived psychosocial benefits of faith: How much is religion***,*** faith***,*** or God a source of strength and comfort to you?***				
Not very much / Somewhat	20/103	Ref	Ref.	Ref
Quite a bit	63/210	1.17 0.84–1.63	1.19 0.85–1.67	1.18 0.84–1.66
A great deal	279/1021	0.83 0.63–1.09	0.88 0.66–1.16	0.86 0.65–1.13
**Religious service attendance: How often do you attend religious or faith services during the year?**				
Never	46/137	Ref	Ref	Ref
Holidays only	109/420	**0.60** **0.42–0.85**	**0.61** **0.43–0.87**	**0.61** **0.43–0.88**
Once per week	106/405	**0.54** **0.38–0.78**	**0.62** **0.43–0.90**	**0.65** **0.45–0.95**
≥ Once per week	104/385	**0.55** **0.38–0.79**	**0.62** **0.42–0.90**	**0.60** **0.41–0.89**

HR^1^: Cox proportional hazards adjusted for age at breast cancer diagnosis, race and time from cohort enrollment to diagnosis

HR^2^: Cox proportional hazards adjusted as in model 1 with the addition of income, education, marital status, insurance

HR^3^: Cox proportional hazards adjusted as in model 2 with the addition of HER2, tumor ER status, tumor PR status

### All-cause mortality among women with breast cancer stratified by race

The results from the race-stratified analysis were similar to those for the population overall, albeit there were minor differences (data not shown). For instance, among White women, we observed a reduced hazard (HR = 0.65, CI: 0.46–0.91) of dying if one has two or more friends/relatives compared to those with one or less, after controlling for age at diagnosis, time from cohort enrollment to diagnosis, income, education, marital status, insurance, addition of HER2, tumor ER status, and tumor PR status; among Black women, this association was weaker and not statistically significant (HR = 0.88, CI: 0.71–1.09). There was a significant association between spirituality and survival among Black women but not among White women. Black women who were very spiritual had a 43% increased hazard of death compared to those who were not spiritual (HR = 1.43, CI: 1.07–1.90) adjusting for age at diagnosis, time from cohort enrollment to diagnosis, income, education, marital status and insurance. For the White women, there was a 32% reduced hazard of death although this was not statistically significant (HR = 0.68, CI: 0.41–1.13), adjusting for age at diagnosis, time from cohort enrollment to diagnosis, income, education, marital status and insurance. Attendance at religious meetings was found to be associated among Black women and not among White women. Among the Black women in the study, there was a reduced hazard of death for those who attended religious services on holidays only (HR = 0.46, CI: 0.28–0.76), once per week (HR = 0.48, CI: 0.29–0.80), more than once per week (HR = 0.43, CI: 0.25–0.72) compared to those who did not attend religious meetings/services adjusting for age at diagnosis, time from cohort enrollment to diagnosis, income, education, marital status, insurance, HER2, tumor ER status, and tumor PR status.

### Breast cancer specific mortality

In the BC-specific analysis (Table 4), out of 365 deaths overall, 218 were BC deaths. Table 4 below gives estimates of the association of social support and religiosity with BC-specific mortality.

**Table 4:** Association between social support characteristics and BC-specific and survival in the whole sample

Psychosocial Variables	All Women with Breast cancer			
	Deaths	HR^1^ (95% CI)	HR^2^ (95% CI)	HR^3^ (95% CI)
**Emotional support: How many close friends or relatives would help you with your emotional problems or feelings if you need it?**				
0 to 1	41/199	Ref	Ref	Ref
2 or more	158/199	**0.77** **0.61–0.96**	0.81 0.65–1.03	0.82 0.65–1.03
**Instrumental support: How many people could you ask for help in an emergency or with lending you money?**				
None to 1	55/99	Ref.	Ref.	Ref
2+	44/99	**0.74** **0.55–0.99**	0.80 0.59–1.07	0.78 0.58–1.05
**Perceived spirituality: How spiritual or religious do you consider yourself to be?**				
Not at all/slightly	133/196	Ref.	Ref.	Ref
Fairly	42/196	0.88 0.63–1.21	0.95 0.68–1.32	0.97 0.70–1.36
Very	21/196	1.42 1.05–1.91	1.38 1.02–1.86	1.34 0.99–1.82
***Perceived psychosocial benefits of faith: How much is religion***,*** faith***,*** or God a source of strength and comfort to you?***				
Not very much / Somewhat	161/198	Ref	Ref	Ref
Quite a bit	25/198	1.26 0.82–1.92	1.27 0.83–1.94	1.28 0.84–1.96
A great deal	12/198	1.05 0.72–1.54	1.13 0.77–1.66	1.12 0.76–1.65
**Religious service attendance: How often do you attend religious or faith services during the year?**				
Never		Ref	Ref	Ref
Holidays only	28/199 54/199	**0.50** **0.32–0.78**	**0.53** **0.34–0.82**	**0.53** **0.34–0.84**
Once per week	60/199	**0.51** **0.33–0.80**	**0.61** **0.39–0.97**	0.65 0.41–1.03
≥ Once per week	57/199	**0.53** **0.34–0.84**	**0.62** **0.39–0.99**	**0.60** **0.37–0.98**

HR^1^: Cox proportional hazards adjusted for age at breast cancer diagnosis, race and time from cohort enrollment to diagnosis

HR^2^: Cox proportional hazards adjusted as in model 1 with the addition of income, education, marital status, insurance

HR^3^: Cox proportional hazards adjusted as in model 2 with the addition of HER2, tumor ER status, tumor PR status

Based on the best fit model (Model 3) using the likelihood ratio test, there was no association between number of people offering emotional support, instrumental support, perceived spirituality and psychosocial benefit of faith (religion, prayer and God as a source of comfort and strength) with survival adjusting for age at BC diagnosis, time from cohort enrollment to diagnosis, income, education, marital status, insurance, HER2, tumor ER status, and tumor PR status. However, attending religious services or faith-based meetings on holidays (HR = 0.53, CI: 0.34–0.84), or more than once per week (HR = 0.60, CI: 0.37–0.98) was associated with reduced hazard of death from BC compared to not attending religious meetings.

## Discussion

This study shows that psychosocial and religiosity factors such as social support and regular religious service attendance were associated with improved survival of women with BC. Specifically, we found that compared to having ≤ 1, having 2 or more friends or relatives for emotional support was associated with a significant 20% reduced hazard of all-cause mortality adjusting for other covariates. Regarding instrumental support which includes having people to ask for help in emergencies or for lending money, those with 2 or more friends or relatives also had reduced hazards of all dying compared to those with only 1 or none. Similarly, women who attended religious services on holidays, once per week and more than once per week had a 39%, 35% and 40% reduced hazards, respectively, of dying compared to those who never attended religious services at all. However, the study did not observe associations between perceived spirituality or perceived faith/religion as source of strength and comfort and survival of women with BC.

Our observation that women with BC have a higher chance of survival if they have more friends or relatives who are able to help them in this journey through instrumental and emotional support is consistent with the results of a study conducted in a large, pooled cohort [9], that found that larger social networks were associated with better BC-specific and overall survival. This suggests that social support is an important aspect in women’s life as the network of family, friends, and others provides practical and emotional assistance. This finding is consistent with what other studies have found [8], in which increased contact with friends/family post-diagnosis of cancer was associated with lower risk of death. Greater social support, and this may increase the likelihood of survival after a diagnosis of BC by enhancing coping skills, providing emotional support and encouragement in taking the medication [10, 23]. Our study has found that women with BC who have larger networks which include friends and family members have a better survival compared to those with 1 or fewer. However, it’s important to note that while social support can have a positive impact on the BC experience, survival outcomes are influenced by a complex interplay of biological, clinical, and psychosocial factors. Women with BC are therefore encouraged to seek and accept support from their social network and to communicate openly with healthcare providers about their needs (Kim et al., 2015).

In our study, we did not find any association between women who were very spiritual and those who were not spiritual and all-cause mortality. Results from our study on spirituality were self-reported and not really an objective measure of religiosity hence this could have been confounded by other unknown variables, or it could have been found by chance. Contrary to previous results reporting significant associations between spirituality and survival [8, 24], our study found no association between spirituality and all-cause mortality, despite the observed association with religious attendance. Religion as a source of strength and comfort did not yield any association with survival, this finding is similar to another study conducted in the US which found that in the US population of low SES, among Black and White participants, comfort from religion had no significant association with reduced end-stage kidney disease [25]. For attendance in religious services, it has been established that those who attended more of such meetings have better survival compared to those who do not attend religious services. This finding is consistent with a study conducted in California US [26] which reported that religious engagement and religious activity help in decreasing the risk of mortality in seventh day Adventists. This could be due to the comfort these women with BC find in the word of God, and the encouragements they receive from the religious meetings. However, we noted in our study that most of these associations were not significant upon adjusting for clinical characteristics such as cancer stage.

When stratified by race, we found White women who had two or more friends/relatives having reduced hazards of death compared to those with one or less social support, but this did not apply to Black women. These findings have been substantiated by a qualitative study in which white women indicated that having other cancer survivors in their network helped to cope with their social support needs but this was not the case with Black women [27]. However, for spirituality we found no association in the white women, but we had an increased hazard of death in the spiritual Black women compared to those who were not spiritual.

In summary, psychosocial support such as social, spiritual and emotional support has been found to be some of the factors that improve survival among individuals with chronic diseases [28, 29] as this helps in decreasing psychological stress [9]. Women with BC also receive encouragements as well as emotional support from friends and family. To our knowledge, studies that have focused on the association between social support, religiosity, and survival of women with BC in a population of low social economic status such as in southeastern community of the United States are not available. While social support has been found to be associated with survival in our study, other studies have further found that it also influence various aspects of a woman’s cancer journey, which include their psychological well-being, treatment adherence, and overall quality of life [30, 31]. Much of the work that has been conducted has been in the general population of women with BC. Our work has delved into understanding if there exist an association between social support and religiosity with survival of women with BC in areas with low social economic status.

### Strengths and limitations

One of the strengths of this study is the large sample size obtained in undeserved population of the southeastern of US and the long follow up period which is ideal for ascertainment of the effect of social support on survival of women with BC. In addition, there was complete ascertainment of the death status of women with BC in the cohort via national death index. Other strengths include the detailed information collected by self-report on social support and religiosity. However, one of the limitations of this study was that the SCCS participants included in the study may not be representative of the general US population. This is because the women with BC were recruited at community health centers hence generalizability is limited to populations of low social economic status. Further, all the exposures were measured/assessed at baseline before cancer diagnosis hence these exposures may have changed after cancer diagnosis.

## Conclusions

Women diagnosed with breast cancer who have a large social network through their friends and families, and through their participation in religious services, have improved survival compared to those who have little/no social support. Further, enhanced social support could be an important piece of post-BC life for women and may improve quality of life. In this regard, women with BC should be encouraged to form support groups through which they will be encouraging one another and give moral support to each other. Results from this study have indicated that there is an association between religiosity and survival, particularly among Black women. Based on these results, we recommend that such studies be conducted in the African setting where religion is very dominant. It would be interesting to see if there could be any link between women living with BC and survival outcomes.

## Electronic supplementary material

Below is the link to the electronic supplementary material.

Supplementary Material 1

## Data Availability

The data that support the findings of this study are not openly available due to reasons of sensitivity and are available from the principal investigator (SCCS) upon reasonable request. Data are in controlled access data storage at Vanderbilt University under the southern community cohort study https://www.southerncommunitystudy.org/.

## Abbreviations

BC: Breast cancer
CI: Confidence interval
ER: Estrogen receptor
HR: Hazards ratio
PR: Progesterone receptor
QoL: Quality of life
SCCS: Southern community cohort study
SES: Socioeconomic status
US: United states
VIF: Variance Inflation Factor
