# Supplementary material for: Association of social support and religiosity with survival among women with breast cancer in a low-income population in the Southeastern United States
Source: BMC Public Health. 2025 Feb 20;25:708. doi: 10.1186/s12889-025-21908-z (PMC11844068; doi:10.1186/s12889-025-21908-z)
Supplement: Supplementary file 1 — Supplementary Material 1 [file 12889_2025_21908_MOESM1_ESM.pdf]

MARKING INSTRUCTIONS
Please use a No. 2 pencil or black or blue ink only.
Print legible numbers and capital block letters in the boxes.

Correct Numbers and Letters 1 2 3 A B C

Correct Mark ■ X

Incorrect Marks ✓ ▬ ·

CONTACT INFORMATION

Name: ____________________

Home Address: ____________________

City: ____________________

State: __________ **Zip Code:** __________

Mailing Address: ____________________
(if different from home address)

Telephone numbers:
HOME ( ) -
WORK ( ) - EXT.
CELL ( ) -

e-Mail Address: ____________________

SAMPLE

GENERAL BACKGROUND INFORMATION

1. What is your date of birth?
Month Day Year

2. What is your sex? ☐ Male ☐ Female

3. How much do you weigh? Pounds

4. What was your weight at age 21? Pounds

5. What is the most you have ever weighed, not counting weight during pregnancy? Pounds

6. How tall are you? Feet Inches

7. Which of the following describes your race or ethnic background? *(Mark all that apply)*
- ☐ White
- ☐ Black/African-American
- ☐ Hispanic/Latino
- ☐ Asian or Pacific Islander
- ☐ American Indian or Alaska Native
- ☐ Other racial or ethnic group

8. What is the highest grade or level of education you have completed? *(Mark the highest)*
- ☐ Less than 9 years
- ☐ 9-11 years
- ☐ Completed high school or GED
- ☐ Vocational, technical, or business training
- ☐ Some college or Junior college
- ☐ Graduated from college
- ☐ Graduate school (up to and including a Master's degree)
- ☐ Graduate school beyond a Master's degree (including doctors, dentists, lawyers, PhDs)

GENERAL BACKGROUND INFORMATION

9. Which of the following describes your total household income last year?

- ☐ Less than $15,000
- ☐ $15,000 - $24,999
- ☐ $25,000 - $49,999
- ☐ $50,000 - $99,999
- ☐ $100,000 or more

10. Where were you born?

- ☐ In the U.S.A. → **which state?** ☐☐
- ☐ Outside the U.S.A.

11. Have you ever lived in a rural or farming community, or on a farm?

- ☐ Yes → **For how many years did you live in a rural or farming community, or on a farm?** *[If less than one year, enter 00.]* **Years** ☐☐
- ☐ No

12. How many full brothers do you have? (Include any that may have died): ☐☐

13. How many full sisters do you have? (Include any that may have died): ☐☐

14. Are you a twin or a triplet (or quadruplet)?

- ☐ Yes
- ☐ No

15. Which of the following describes your current marital status?

- ☐ Married, or living as married with a partner
- ☐ Separated or divorced
- ☐ Widowed
- ☐ Single – never been married

16. How many people live in your household now, including yourself? ☐☐

TOBACCO USE

17. Whether or not you smoke, about HOW MANY HOURS PER WEEK are you around the cigarette smoke of OTHER PEOPLE?

In your home: **Hours per week** ☐☐☐

In other indoor places (including work): **Hours per week** ☐☐☐

18. Have you smoked at least 100 cigarettes in your entire life?

- ☐ Yes → At what age did you start to smoke? ☐☐
- ☐ No → Go to question 22.

19. Do you smoke cigarettes now?

- ☐ Yes
- ☐ No → **At what age did you quit?** ☐☐

20. What is the average number of cigarettes that you smoke (or used to smoke, if you have quit) in one day? ☐☐☐

21. Are the cigarettes you usually smoke (or used to smoke, if you have quit) menthol?

- ☐ Yes
- ☐ No

22. In your entire life:

		**How much do you use?** *[If less than one, enter 00.]*	**For how many years have you used this tobacco product?** *[If less than one year, enter 00.]*
**Have you smoked at least 20 CIGARS?** ☐ Yes → ☐ No	**Do you STILL smoke cigars?** ☐ Yes → ☐ No	☐☐ cigars per day	☐☐ years
**Have you smoked at least 20 PIPESFULL OF TOBACCO?** ☐ Yes → ☐ No	**Do you STILL smoke pipes?** ☐ Yes → ☐ No	☐☐ pipesfull per day	☐☐ years
**Have you used at least 1 bag of CHEWING TOBACCO?** ☐ Yes → ☐ No	**Do you STILL use chewing tobacco?** ☐ Yes → ☐ No	☐☐ plugs per day	☐☐ years

SAMPLE

TOBACCO USE

22. In your entire life:

Have you used at least 1 can of SNUFF?
☐ Yes → **Do you STILL use snuff?** ☐ Yes →
☐ No ☐ No

How much do you use? *[If less than one, enter 00.]*
☐☐ dips per day

For how many years have you used this tobacco product? *[If less than one year, enter 00.]*
☐☐ years

MEDICAL HISTORY

23. Has a doctor ever told you that you have had any of the following conditions, or have you ever been treated for any of the following conditions?

	Mark if "Yes"	WHAT WAS YOUR AGE AT FIRST DIAGNOSIS
**High blood pressure** (not during pregnancy)	☐	☐☐
**Diabetes or high blood sugar** (not during pregnancy)	☐	☐☐
**Heart attack or coronary artery bypass surgery**	☐	☐☐
**High cholesterol**	☐	☐☐
**Stroke/mini-stroke/transient ischemic attack (TIA)**	☐	☐☐
**Hepatitis**	☐	☐☐
**(If yes, which type?)** → *(Mark all that apply)* ☐ A ☐ B ☐ C ☐ Don't know ☐ Other type (Specify) ______		
**Emphysema or chronic bronchitis**	☐	☐☐
**Tuberculosis (TB)**	☐	☐☐
**Asthma**	☐	☐☐
**Hayfever, skin allergy, food allergy, or other allergy**	☐	☐☐
**Sickle cell disease**	☐	☐☐
**Polyps in colon or rectum**	☐	☐☐

	Mark if "Yes"	WHAT WAS YOUR AGE AT FIRST DIAGNOSIS
**Heartburn or acid reflux**	☐	☐☐
**Ulcer** (stomach or duodenal)	☐	☐☐
**Crohn's disease or ulcerative colitis**	☐	☐☐
**Glaucoma**	☐	☐☐
**Cataracts**	☐	☐☐
**HIV/AIDS**	☐	☐☐
**Parkinson's disease**	☐	☐☐
**Lupus** (systemic lupus erythematosis/SLE)	☐	☐☐
**Multiple sclerosis**	☐	☐☐
**Depression**	☐	☐☐
**Arthritis**	☐	☐☐
**Non-cancerous cyst in the breast**	☐	☐☐
**Fibroids in the uterus (womb)**	☐	☐☐

SAMPLE

MEDICAL HISTORY

23. Has a doctor ever told you that you have had any of the following conditions, or have you ever been treated for any of the following conditions?

CANCER of any kind ☐ Yes → ☐ No

If yes, what type of cancer?		WHAT WAS YOUR AGE AT FIRST DIAGNOSIS?
☐ Breast		☐☐
☐ Prostate		☐☐
☐ Colorectal		☐☐
☐ Lung		☐☐
☐ Uterine/endometrial		☐☐
☐ Cervical		☐☐
☐ Non-melanoma skin		☐☐
☐ Other (specify):		☐☐
☐ Other (specify):		☐☐

SAMPLE

FAMILY HISTORY OF CANCER

24. Has your BIRTH MOTHER ever had cancer? ☐ Yes ☐ No ☐ Don't Know

If yes, what kind of cancer did your BIRTH MOTHER have?		Was she diagnosed before she was 50 years old?		
☐ Breast	→	☐ Yes	☐ No	☐ Don't Know
☐ Colorectal	→	☐ Yes	☐ No	☐ Don't Know
☐ Lung	→	☐ Yes	☐ No	☐ Don't Know
☐ Uterine/endometrial	→	☐ Yes	☐ No	☐ Don't Know
☐ Cervical	→	☐ Yes	☐ No	☐ Don't Know
☐ Ovarian	→	☐ Yes	☐ No	☐ Don't Know
☐ Stomach	→	☐ Yes	☐ No	☐ Don't Know
☐ Other (specify):	→	☐ Yes	☐ No	☐ Don't Know
☐ Other (specify):	→	☐ Yes	☐ No	☐ Don't Know

25. If you have any FULL SISTERS, have any ever had cancer? ☐ Yes ☐ No ☐ Don't Know

If yes, what kind of cancer did she (or they) have?	How many sisters had this cancer?	Were any sisters diagnosed before age 50?
☐ Breast	→ ☐☐ sisters	☐ Yes ☐ No ☐ Don't Know
☐ Colorectal	→ ☐☐ sisters	☐ Yes ☐ No ☐ Don't Know
☐ Lung	→ ☐☐ sisters	☐ Yes ☐ No ☐ Don't Know
☐ Uterine/endometrial	→ ☐☐ sisters	☐ Yes ☐ No ☐ Don't Know
☐ Cervical	→ ☐☐ sisters	☐ Yes ☐ No ☐ Don't Know
☐ Ovarian	→ ☐☐ sisters	☐ Yes ☐ No ☐ Don't Know
☐ Stomach	→ ☐☐ sisters	☐ Yes ☐ No ☐ Don't Know
☐ Other (specify): ______	→ ☐☐ sisters	☐ Yes ☐ No ☐ Don't Know
☐ Other (specify): ______	→ ☐☐ sisters	☐ Yes ☐ No ☐ Don't Know

26. Has your BIRTH FATHER ever had cancer? ☐ Yes ☐ No ☐ Don't Know

If yes, what kind of cancer did your BIRTH FATHER have?	Was he diagnosed before he was 50 years old?
☐ Prostate	→ ☐ Yes ☐ No ☐ Don't Know
☐ Colorectal	→ ☐ Yes ☐ No ☐ Don't Know
☐ Lung	→ ☐ Yes ☐ No ☐ Don't Know
☐ Stomach	→ ☐ Yes ☐ No ☐ Don't Know
☐ Throat/pharynx	→ ☐ Yes ☐ No ☐ Don't Know
☐ Other (specify): ______	→ ☐ Yes ☐ No ☐ Don't Know
☐ Other (specify): ______	→ ☐ Yes ☐ No ☐ Don't Know

SAMPLE

FAMILY HISTORY OF CANCER

27. If you have any FULL BROTHERS, have any ever had cancer? ☐ Yes ☐ No ☐ Don't Know

If yes, what kind of cancer did he (or they) have?	How many brothers had this cancer?	Were any brothers diagnosed before age 50?
☐ Prostate	→ ☐☐ brothers	☐ Yes ☐ No ☐ Don't Know
☐ Colorectal	→ ☐☐ brothers	☐ Yes ☐ No ☐ Don't Know
☐ Lung	→ ☐☐ brothers	☐ Yes ☐ No ☐ Don't Know
☐ Stomach	→ ☐☐ brothers	☐ Yes ☐ No ☐ Don't Know
☐ Throat/pharynx	→ ☐☐ brothers	☐ Yes ☐ No ☐ Don't Know
☐ Other (specify): ______	→ ☐☐ brothers	☐ Yes ☐ No ☐ Don't Know
☐ Other (specify): ______	→ ☐☐ brothers	☐ Yes ☐ No ☐ Don't Know

FAMILY HISTORY OF HEART DISEASE

28. Have any of the following family members had a heart attack or had coronary artery bypass surgery?

	Mark if "Yes"	If yes, at what age did this first occur?		
Birth Mother	☐	☐ ≤55	☐ 56-64	☐ 65+
Birth Father	☐	☐ ≤55	☐ 56-64	☐ 65+
Full Sister(s)	☐	☐ ≤55	☐ 56-64	☐ 65+
Full Brother(s)	☐	☐ ≤55	☐ 56-64	☐ 65+

[Mark the youngest age that applies if more than one full brother or full sister has had these heart problems.]

FAMILY HISTORY OF DIABETES

29. Have any of the following family members had diabetes or high blood sugar?

	Mark if "Yes"	If yes, at what age was this first diagnosed?		
Birth Mother	☐	☐ ≤19	☐ 20-64	☐ 65+
Birth Father	☐	☐ ≤19	☐ 20-64	☐ 65+
Full Sister(s)	☐	☐ ≤19	☐ 20-64	☐ 65+
Full Brother(s)	☐	☐ ≤19	☐ 20-64	☐ 65+

[Mark the youngest age that applies if more than one full brother or full sister has had diabetes.]

ONLY MEN SHOULD COMPLETE QUESTION 30.

30. Has a doctor ever told you that you have an enlarged prostate (also known as BPH or benign prostatic hyperplasia)? ☐ No ☐ Yes → **Have you ever had surgery for this?** ☐ Yes ☐ No

ONLY WOMEN SHOULD COMPLETE QUESTIONS 31 TO 39.

31. How old were you when you had your first menstrual period? ☐☐

SAMPLE

32. Have you ever been pregnant?

☐ Yes → **How many times?** ☐☐

[Include ALL pregnancies, even those that did not result in a birth.]

☐ No → **Go to question 37.**

33. Did you develop diabetes or high blood sugar during any of your pregnancies?

☐ Yes ☐ No

34. How many of your pregnancies have resulted in a live birth?

☐☐ pregnancies

35. If you ever gave birth, how old were you at the time you:

FIRST gave birth? ☐☐ **LAST gave birth?** ☐☐

36. How many months TOTAL (counting all of your pregnancies) did you breast feed?

☐☐ Months

37. Did you ever have your tubes tied (a tubal ligation)?

☐ Yes → **How old were you when you had your tubes tied?** ☐☐

☐ No

38. Have you been through menopause, or have your menstrual periods stopped for at least six months?

☐ Yes → **How old were you when your NATURAL menstrual periods stopped?** ☐☐

☐ No

Which of the following is the reason your periods stopped?

☐ Natural menopause

☐ Radiation, chemotherapy, or medication

☐ Surgery that removed your uterus (womb) or ovaries

☐ Other reason

39. Have you had your uterus (womb) or any ovaries removed?

☐ Yes → **Please mark ALL that were removed:**

☐ No

☐ **Uterus** ☐☐ At what age?

☐ **One ovary** ☐☐ At what age?

☐ **Two ovaries** ☐☐ At what age?

MEDICATION USE

40. In the past year, have you taken any of the following medications REGULARLY? By REGULARLY, we mean AT LEAST TWO TIMES PER WEEK FOR ONE MONTH OR MORE.

	No	Yes	How many years have you taken this type of medication REGULARLY? *[If less than one year, enter 00.]*	When you took this regularly, what is the average number of pills you took per week?
**Low-dose aspirin, baby aspirin, or half-tablets of aspirin** (to prevent heart disease or strokes)	☐	☐ ▶	☐☐ years	☐☐ pills
**Regular aspirin** (such as Anacin, Bayer, Bufferin, Excedrin, etc.)	☐	☐ ▶	☐☐ years	☐☐ pills
**Acetaminophen** (such as Tylenol)	☐	☐ ▶	☐☐ years	☐☐ pills
**The prescription drugs Celebrex, Vioxx, or Bextra**	☐	☐ ▶	☐☐ years	☐☐ pills
**Advil, Motrin, Aleve, Ibuprofen,** or other over-the-counter pain relievers	☐	☐ ▶	☐☐ years	☐☐ pills

41. How many times have you filled a prescription for antibiotics for yourself in the past year? ☐☐☐ times

SAMPLE

42. Are you currently taking prescription medicine to lower your blood pressure?

- ☐ Yes → **Please mark ALL that you take:**
  - ☐ Accupril
  - ☐ Adalat or Procardia
  - ☐ Aldactone
  - ☐ Altace
  - ☐ Calan, Isoptin or Verelan
  - ☐ Capoten
  - ☐ Cardizem or Tiazac
  - ☐ Catapres
  - ☐ Cozaar
  - ☐ Diovan
  - ☐ Diovan HCT
  - ☐ Esidrix or Hydrodiuril (HCT)
  - ☐ Lasix
  - ☐ Lopressor or Toprol-XL
  - ☐ Lotrel
  - ☐ Norvasc
  - ☐ Prinivil or Zestril
  - ☐ Tenormin
  - ☐ Other (specify): ______
- ☐ No

43. Are you currently taking prescription medicine, including insulin, to lower your blood sugar?

- ☐ Yes → **Please mark ALL that you take:**
  - ☐ Insulin (any type)
  - ☐ Actos
  - ☐ Amaryl
  - ☐ Avandamet
  - ☐ Avandia
  - ☐ Glipzide
  - ☐ Glucophage
  - ☐ Glucotrol
  - ☐ Glucovance
  - ☐ Glyburide
  - ☐ Metformin
  - ☐ Other (specify): ______
- ☐ No

During the past three months, how often did you take your diabetes medication as instructed by your doctor?

- ☐ All the time
- ☐ Most of the time
- ☐ Sometimes
- ☐ Rarely
- ☐ Never

During the past three months, how often did you test your own blood sugar (not including at the doctor's office)?

- ☐ Less than once per week
- ☐ 1-6 times per week
- ☐ Once per day
- ☐ Twice per day
- ☐ Three times per day
- ☐ Four or more times per day

44. Are you currently taking prescription medicine to lower your cholesterol?

- ☐ Yes → **Please mark ALL that you take:**
  - ☐ Crestor
  - ☐ Gemfibrozil
  - ☐ Lescol
  - ☐ Lipitor
  - ☐ Lopid
  - ☐ Pravachol
  - ☐ Zetia
  - ☐ Zocor
  - ☐ Other (specify): ______
- ☐ No

→ **For how many years have you taken this medication?** *[Enter the longest number of years if you are currently taking more than one medicine.]* ☐☐

45. Are you currently taking any anti-depressant or anti-anxiety prescription medication?

- ☐ Yes → **Please mark ALL that you take:**
  - ☐ Ambien
  - ☐ Buspar
  - ☐ Celexa
  - ☐ Dilantin
  - ☐ Effexor
  - ☐ Lexapro
  - ☐ Paxil
  - ☐ Prozac
  - ☐ Remeron
  - ☐ Risperdal
  - ☐ Seroquel
  - ☐ Topamax
  - ☐ Wellbutrin
  - ☐ Xanax
  - ☐ Zoloft
  - ☐ Other (specify): ______
- ☐ No

ONLY WOMEN SHOULD COMPLETE QUESTIONS 46 TO 49.

46. Have you EVER USED female hormone replacement therapy that contains estrogen and/or progesterone, for menopause? (this includes pills, patches, creams, and injections)

- ☐ Yes
- ☐ No → Go to question 49.

47. Are you currently taking hormone replacement therapy?

- ☐ No → **How many years ago did you LAST take hormone replacement therapy?** *[If less than one year ago, enter 00.]* ☐☐
- ☐ Yes → **Please mark ALL that you take:**
  - ☐ Premarin
  - ☐ Prempro
  - ☐ Estrogen
  - ☐ Estradiol
  - ☐ Estracep
  - ☐ Estratest
  - ☐ Prometrium
  - ☐ Vivelle
  - ☐ Cenestin
  - ☐ Provera
  - ☐ Climara
  - ☐ Activella
  - ☐ Other (specify): ______

48. How old were you when you first started taking hormone replacement therapy? ☐☐

49. Did you ever use birth control pills?

- ☐ Yes → **How old were you when you first started using birth control pills?** ☐☐ → **For how many years have you used birth control pills, IN TOTAL?** *[If less than one year, enter 00.]* ☐☐
- ☐ No

SAMPLE

50. How many hours do you typically sleep in a 24-hour period?

	Hours
On weekdays →	
On weekends →	

51. How much TIME PER DAY do you typically spend:

	Hours	Minutes
Sitting in a car or bus		
Sitting at work		
Watching TV or seeing movies		
**Using a computer at home** *(such as email, internet, games)*		
**Other sitting activities** *(such as sitting at meals, talking on the phone, reading, playing cards, or sewing)*		

52. How much TIME PER DAY do you typically spend:

	Hours	Minutes
**Walking slowly** *(such as moving around, walking at work, walking the dog, or for light exercise)*		
**Walking fast** *(such as climbing stairs, walking fast to go places, or for exercise)*		

53. How much TIME PER DAY do you typically spend doing:

	On weekdays		On weekends	
	Hours	Minutes	Hours	Minutes
**Light work** *(such as standing at work, light office work, shopping, cooking, or child and elderly care)*				
**Moderate Work** *(such as manufacturing work, shop work, cleaning house, gardening, mowing the lawn, or home repair)*				
**Strenuous Physical Work** *(such as moving furniture, loading or unloading trucks, construction work, farming, or other hard labor)*				

54. How much TIME PER WEEK do you typically spend doing:

	Hours	Minutes
**Moderate Sports** *(such as bowling, dancing, golfing, or softball)*		
**Vigorous Sports** *(such as jogging, aerobics, bicycling, tennis, swimming, weight lifting, or basketball)*		

55. Thinking back to when you were IN YOUR THIRTIES, about how much TIME PER DAY did you typically spend doing:

	Hours	Minutes
**Light work** *(such as standing at work, light office work, shopping, cooking, child and elderly care)*		
**Moderate Work** *(such as manufacturing work, shop work, cleaning house, gardening, mowing the lawn, or home repair)*		
**Strenuous Physical Work** *(such as moving furniture, loading or unloading trucks, construction work, farming, or other hard labor)*		

SAMPLE

56. **Thinking back to when you were IN YOUR THIRTIES, about how much TIME PER WEEK did you typically spend doing:**

	Hours	Minutes		Hours	Minutes
**Moderate Sports** *(such as bowling, dancing, golfing, or softball)*			**Vigorous Sports** *(such as jogging, aerobics, bicycling, tennis, swimming, weight lifting, or basketball)*		

EMOTIONAL WELL-BEING AND SUPPORT

57. **This question asks about HOW YOU WERE FEELING DURING THE PAST WEEK.**

	Rarely or none of the time	Some of the time	Much of the time	Most or all of the time
I was happy.	☐	☐	☐	☐
I felt lonely.	☐	☐	☐	☐
I could not get "going".	☐	☐	☐	☐
I was bothered by things that usually don't bother me.	☐	☐	☐	☐
I had trouble keeping my mind on what I was doing.	☐	☐	☐	☐
I felt depressed.	☐	☐	☐	☐
I felt that everything I did was an effort.	☐	☐	☐	☐
I felt hopeful about the future.	☐	☐	☐	☐
I felt fearful.	☐	☐	☐	☐
My sleep was restless.	☐	☐	☐	☐

58. **In the PAST MONTH, how often have you felt that:**

	Rarely or none of the time	Some of the time	Much of the time	Most or all of the time
you were unable to control the important things in your life ?	☐	☐	☐	☐
difficulties were piling up so high that you could not overcome them?	☐	☐	☐	☐

59. **How many close friends or relatives would help you with your emotional problems or feelings if you needed it?** ☐☐

60. **How many people could you ask for help in an emergency or with lending you money?** ☐☐

RELIGION AND SPIRITUALITY

61. **How often do you attend religious or faith services during the year?**

- ☐ Never
- ☐ On major holy days or holidays only
- ☐ More than 4 times per year, but not every week
- ☐ Once per week
- ☐ More than once per week

62. **How spiritual or religious do you consider yourself to be:**

- ☐ Very
- ☐ Fairly
- ☐ Slightly
- ☐ Not at all

63. **How much is religion, faith, or God a source of strength and comfort to you?**

- ☐ Not very much
- ☐ Somewhat
- ☐ Quite a bit
- ☐ A great deal

SAMPLE

64. Are you covered by any type of health insurance, including private insurance, Medicare or Medicaid?

☐ Yes → **Which type?:** *(Mark all that apply)*
- ☐ Medicaid or medical assistance
- ☐ Medicare
- ☐ Private insurance or employer insurance (for example, insurance through your job, like Blue Cross or another private insurance company)
- ☐ CHAMPUS/Tricare/CHAMPVA/V.A./military
- ☐ Other type of health insurance

☐ No

65. When was your *last* visit to a doctor or other medical person? This could be for a routine check-up, because you were sick, or simply to get health advice.

years ago ☐☐ OR months ago ☐☐

66. When was your *last* visit to a dentist?

years ago ☐☐ OR months ago ☐☐

67. Did you visit an "alternative medicine" (or "complementary medicine") practitioner during the past year? (such as a chiropractor, massage therapist, herbalist, acupuncturist, traditional healer, etc.)

☐ Yes ☐ No

CANCER SCREENING EXAMS

68. If you are a woman, in the past year, how many times did you practice BREAST SELF-EXAMINATION (feeling your own breasts for lumps)? ☐☐ **times**

69. Have you ever had a:

WOMEN

Pap smear? (test for cervical cancer)

☐ Yes → **When was your last Pap Smear?**
years ago ☐☐ *[If less than one year ago, enter 00.]*

☐ No

Mammogram? (x-ray to check for breast cancer)

☐ Yes → **When was your last Mammogram?**
years ago ☐☐ *[If less than one year ago, enter 00.]*

☐ No

Sigmoidoscopy? (short tube inserted into rectum while you are awake and unsedated to check for colon or rectal cancer)

☐ Yes → **When was your last Sigmoidoscopy?**
years ago ☐☐ *[If less than one year ago, enter 00.]*

☐ No

Colonoscopy? (long tube inserted into rectum after you are sedated or put to sleep to check for colon or rectal cancer)

☐ Yes → **When was your last Colonoscopy?**
years ago ☐☐ *[If less than one year ago, enter 00.]*

☐ No

SAMPLE

2332 28-2 576

MEN

Digital Rectal Exam? (doctor's finger inserted into your rectum to feel for prostate cancer)

☐ Yes → **When was your last Digital Rectal Exam?**
☐ No

years ago ☐☐ *[If less than one year ago, enter 00.]*

PSA blood test? (blood test to check for prostate cancer)

☐ Yes → **When was your last PSA blood test?**
☐ No

years ago ☐☐ *[If less than one year ago, enter 00.]*

Sigmoidoscopy? (short tube inserted into rectum while you are awake and unsedated to check for colon or rectal cancer)

☐ Yes → **When was your last Sigmoidoscopy?**
☐ No

years ago ☐☐ *[If less than one year ago, enter 00.]*

Colonoscopy? (long tube inserted into rectum after you are sedated or put to sleep to check for colon or rectal cancer)

☐ Yes → **When was your last Colonoscopy?**
☐ No

years ago ☐☐ *[If less than one year ago, enter 00.]*

70. If you have NEVER had any of the following tests, or if you had any of the following tests MORE THAN 5 YEARS AGO, please mark any of the following reasons why you have not had this test recently. *Mark as many reasons as apply.*

Reasons you have not had this test recently:	WOMEN ANSWER THESE: Mammogram	Pap smear	Colonoscopy/ Sigmoidoscopy	MEN ANSWER THESE: Digital rectal exam	PSA blood test
Your doctor has not recommended this test	☐	☐	☐	☐	☐
You forgot to do it	☐	☐	☐	☐	☐
The fear of finding cancer	☐	☐	☐	☐	☐
You put it off or you're too busy	☐	☐	☐	☐	☐
The embarrassment	☐	☐	☐	☐	☐
The cost	☐	☐	☐	☐	☐
The pain or discomfort that you may experience during this test	☐	☐	☐	☐	☐
None of these reasons apply	☐	☐	☐	☐	☐

ENVIRONMENT IN AND AROUND YOUR HOME

71. How many years have you lived in your current home? ☐☐ **years** *[If less than one year, enter 00.]*

72. How would you describe the outdoor air quality around your home?

☐ Poor
☐ Fair
☐ Good
☐ Excellent

73. How would you describe the indoor air quality in your home?

☐ Poor
☐ Fair
☐ Good
☐ Excellent

SAMPLE

CELLULAR TELEPHONE USE

74. How many hours per week do you spend talking on a cellular phone? ☐☐ **hours** *[If less than one hour, enter 00.]*

SEAT BELT USE

75. When you are in a vehicle, are you more likely to drive or ride? ☐ Drive ☐ Ride

76. How often do you use seat belts

- ☐ All of the time
- ☐ Most of the time
- ☐ Some of the time
- ☐ None of the time

} → **What are the reasons you do not always use seat belts?** *(Mark ALL that apply)*

- ☐ Seatbelts are hard to fasten
- ☐ Seatbelts wrinkle your clothes
- ☐ Seatbelts restrict your movement
- ☐ Seat belts are uncomfortable
- ☐ You forget to put them on
- ☐ If you have an accident, you will not be able to get out
- ☐ You're only going a short distance
- ☐ You're in a rush
- ☐ None of these reasons apply

WORK HISTORY

77. What type of job did you have for the LONGEST PERIOD OF TIME DURING YOUR ADULT LIFE?
(Mark only one.)

- ☐ Laborer, including construction worker
- ☐ Farmer or farm worker
- ☐ Professional/Technician
- ☐ Supervisory position (Manager/Administrator)
- ☐ Sales worker
- ☐ Small business owner
- ☐ Clerical worker
- ☐ Personal service worker
- ☐ Craftsperson
- ☐ Factory worker or machine operator
- ☐ Transportation worker or driver
- ☐ Food preparation or service worker
- ☐ Protective service worker
- ☐ Community/social services worker
- ☐ Educator/Trainer/Librarian
- ☐ Maintenance and repair worker
- ☐ Military officer or enlisted person
- ☐ Housewife
- ☐ I have never worked
- ☐ Some other type of job

78. Are you currently working? ☐ Yes ☐ No
(If yes, which of the following job types do you currently have?)

(Mark only one.)

- ☐ Laborer, including construction worker
- ☐ Farmer or farm worker
- ☐ Professional/Technician
- ☐ Supervisory position (Manager/Administrator)
- ☐ Sales worker
- ☐ Small business owner
- ☐ Clerical worker
- ☐ Personal service worker
- ☐ Craftsperson
- ☐ Factory worker or machine operator
- ☐ Transportation worker or driver
- ☐ Food preparation or service worker
- ☐ Protective service worker
- ☐ Community/social services worker
- ☐ Educator/Trainer/Librarian
- ☐ Maintenance and repair worker
- ☐ Military officer or enlisted person
- ☐ Housewife
- ☐ Some other type of job

SAMPLE

WORK HISTORY

79. Please mark any of the following industries or occupations in which you were employed FOR 10 YEARS OR LONGER. *(Mark ALL that apply.)*

- ☐ Automotive repair
- ☐ Chemical production or use
- ☐ Construction
- ☐ Cotton, wool, or textile processing
- ☐ Dry cleaning
- ☐ Farming
- ☐ Furniture making or woodworking
- ☐ Gasoline refining or redistribution
- ☐ Hairdressing
- ☐ Metal production or processing
- ☐ Military
- ☐ Mining, quarrying, rock crushing, or cement manufacturing
- ☐ Nursing
- ☐ Paint production or use
- ☐ Paper or pulp mill work
- ☐ Pesticide production or use
- ☐ Plastic production or processing
- ☐ Rubber or tire manufacturing
- ☐ Shipyard work
- ☐ None of these

YOUR USUAL EATING HABITS

80. Thinking about your usual eating habits OVER THE PAST YEAR, HOW MANY TIMES PER DAY did you typically eat (or drink):

	**Number of times per day** *[If less than once per day, enter 00.]*		**Number of times per day** *[If less than once per day, enter 00.]*
Meat, sandwiches made with meat, meat in mixed dishes, or eggs	► ☐☐	Vegetables	► ☐☐
Breads, cereals, rice, potatoes, or pastas	► ☐☐	Fruits or fruit juices	► ☐☐
Desserts, candy, cookies, sweets, or salty snacks	► ☐☐	Soft drinks, Kool-Aid, or other sweetened drinks	► ☐☐
Milk, cheese, ice cream, yogurt, or other dairy products	► ☐☐	Water	► ☐☐

81. The next questions ask about your USUAL EATING HABITS DURING THE PAST YEAR. For each food item, mark the box to show how often you usually ate that item. Please mark only one answer per food item.

REMEMBER
If you never ate a certain food, mark the "Never" column, don't leave it blank!

For example, if you normally ate eggs for breakfast on Saturdays and Sundays, you would mark the box for "2-3 times a week" as shown at right:

**Food Items**	**Average use over the past year**								
	Never	Rarely	1/month	2–3/month	1/week	2–3/week	4–6/week	1/day	2+/day
Eggs such as fried, scrambled, boiled, omelets	☐	☐	☐	☐	☐	X	☐	☐	☐

SAMPLE

Food Items	Average use over the past year								
	Never	Rarely	1/month	2–3/month	1/week	2–3/week	4–6/week	1/day	2+/day
**Fruits and Fruit Juices**									
Bananas									
Apples, pears, or apple sauce									
Watermelon, cantaloupe, or honeydew									
Grapes, strawberries, blueberries, raspberries, or blackberries									
Oranges or grapefruit (not including juice)									
Peaches or nectarines									
Other fruits such as pineapple, mixed fruit, fruit salad, raisins, plums, or prunes									
100% orange juice or grapefruit juice (fresh, frozen, or canned)									
100% fruit juice such as apple, grape, etc.									
Fruit flavored drinks such as Kool-Aid, punch, Tang, lemonade, or Sunny Delight									
**Breakfast Foods**									
Bran or high fiber cereals such as All Bran, Raisin Bran, or Shredded Wheat									
Other cold cereals such as Corn Flakes, Cheerios, or Product 19									
Grits									
Oatmeal, cream of wheat, or other hot cereals									
Eggs such as fried, scrambled, hard boiled or omelets									
Bacon or breakfast sausage (include breakfast sandwiches)									
Biscuits									
**Vegetables**									
Broccoli, cabbage, brussels sprouts, or cauliflower									
Mustard greens, turnip greens, collards, or spinach									
Tomatoes (fresh or canned) and tomato or vegetable juice									
Onions									
Carrots or mixed vegetables with carrots (raw or cooked)									
Corn (fresh, canned, or frozen)									

Food Items	Average use over the past year								
	Never	Rarely	1/month	2–3/month	1/week	2–3/week	4–6/week	1/day	2+/day
**Vegetables**									
Green beans or green peas (fresh, canned, or frozen)									
Lettuce, green salad, or tossed salad with lettuce									
Coleslaw or sauerkraut									
Other vegetables such as eggplant, mushrooms, celery, cucumber, squash, or okra									
**Rice, Beans and Potatoes**									
Rice mixed with meat or seafood (such as dirty rice or jambalaya)									
Plain rice or rice mixed with vegetables or beans (include white or brown rice)									
Pork and beans, baked beans, or chili with beans									
Other dried and canned beans such as pinto, white, kidney, black eyed peas, or field peas									
Sweet potatoes or yams									
Fried potatoes (such as french fries, home fries, hash browns, or tater tots)									
Mashed, scalloped, baked, or boiled potatoes									
**Pasta, Pizza and Soup**									
Spaghetti, ravioli, lasagna, or other pasta with tomato or meat sauce									
Macaroni and cheese									
Pizza (home made, frozen, or from a restaurant)									
Soups or chowders (such as tomato, vegetable, noodle, rice, etc.)									
**Meat**									
Fried beef (include chicken fried steak and steak with gravy)									
Hamburgers, cheeseburgers, or sloppy joes									
Other ground beef such as meatloaf, meatballs, or patties									
Beef in mixed dishes such as stew, pot pies, casserole, or stirfry									
Roast beef, steak, or beef barbeque (include frozen dinners and sandwiches)									
Liver, kidneys, chitterlings, ham hocks, pigs feet, or other organ meats									

SAMPLE

Food Items		Never	Rarely	1/month	2–3/month	1/week	2–3/week	4–6/week	1/day	2+/day
		Average use over the past year								
Meat	Fried chicken or chicken nuggets	☐	☐	☐	☐	☐	☐	☐	☐	☐
Meat	Baked, broiled, or boiled chicken or turkey (including frozen dinners and sandwiches)	☐	☐	☐	☐	☐	☐	☐	☐	☐
Meat	Chicken in mixed dishes, casserole, stir-fries, or chicken pot pie	☐	☐	☐	☐	☐	☐	☐	☐	☐
Meat	Roast pork, pork chops, dinner ham, pork spareribs, or pork barbeque	☐	☐	☐	☐	☐	☐	☐	☐	☐
Meat	Canned tuna fish, tuna casserole, or tuna salad (including sandwiches)	☐	☐	☐	☐	☐	☐	☐	☐	☐
Meat	Fried fish, shrimp, or seafood (including sandwiches)	☐	☐	☐	☐	☐	☐	☐	☐	☐
Meat	Broiled or baked fish, shrimp, or seafood (including sandwiches)	☐	☐	☐	☐	☐	☐	☐	☐	☐
Meat	Bologna, salami, or other lunch meats	☐	☐	☐	☐	☐	☐	☐	☐	☐
Meat	Hot dogs or sausage (such as Kielbasa, Italian, Polish, Vienna, etc.) (do not include breakfast sausage)	☐	☐	☐	☐	☐	☐	☐	☐	☐
Meat	Meat substitutes such as veggie-burgers, soy products, or tofu	☐	☐	☐	☐	☐	☐	☐	☐	☐
Breads	White bread, rolls, dinner rolls, buns, or bagels (include sandwiches)	☐	☐	☐	☐	☐	☐	☐	☐	☐
Breads	Dark or whole grain breads (include sandwiches)	☐	☐	☐	☐	☐	☐	☐	☐	☐
Breads	Corn bread, corn muffins, corn tortillas, or hush puppies	☐	☐	☐	☐	☐	☐	☐	☐	☐
Spreads and Dressings	Butter (real butter, not margarine, added to foods such as bread, grits, rice, and vegetables)	☐	☐	☐	☐	☐	☐	☐	☐	☐
Spreads and Dressings	Margarine (added to foods such as breads, grits, rice, and vegetables)	☐	☐	☐	☐	☐	☐	☐	☐	☐
Spreads and Dressings	Regular salad dressing or mayonnaise (added to salads or sandwiches)	☐	☐	☐	☐	☐	☐	☐	☐	☐
Spreads and Dressings	Low fat or reduced fat salad dressing or mayonnaise (added to salads or sandwiches)	☐	☐	☐	☐	☐	☐	☐	☐	☐
Spreads and Dressings	Jelly, jam, preserves, honey, or syrup	☐	☐	☐	☐	☐	☐	☐	☐	☐
Spreads and Dressings	Peanut butter (include sandwiches)	☐	☐	☐	☐	☐	☐	☐	☐	☐
Dairy Foods	Skim milk or buttermilk (include milk on cereal and added to coffee or tea)	☐	☐	☐	☐	☐	☐	☐	☐	☐
Dairy Foods	1% or 2% lowfat milk (include milk on cereal and added to coffee or tea)	☐	☐	☐	☐	☐	☐	☐	☐	☐

Food Items		Never	Rarely	1/month	2–3/month	1/week	2–3/week	4–6/week	1/day	2+/day
		Average use over the past year								
Dairy Foods	Whole milk (include milk on cereal and added to coffee or tea)	☐	☐	☐	☐	☐	☐	☐	☐	☐
Dairy Foods	Cream or whipped cream (include cream added to coffee or tea or on desserts)	☐	☐	☐	☐	☐	☐	☐	☐	☐
Dairy Foods	Cottage cheese or yogurt	☐	☐	☐	☐	☐	☐	☐	☐	☐
Dairy Foods	Other cheese such as American, processed, Swiss, or cheddar	☐	☐	☐	☐	☐	☐	☐	☐	☐
Desserts and Snacks	Ice cream	☐	☐	☐	☐	☐	☐	☐	☐	☐
Desserts and Snacks	Frozen yogurt, ice milk, or sherbet	☐	☐	☐	☐	☐	☐	☐	☐	☐
Desserts and Snacks	Cookies	☐	☐	☐	☐	☐	☐	☐	☐	☐
Desserts and Snacks	Cake	☐	☐	☐	☐	☐	☐	☐	☐	☐
Desserts and Snacks	Baked or fried pies or cobblers	☐	☐	☐	☐	☐	☐	☐	☐	☐
Desserts and Snacks	Doughnuts, sweet rolls, pastry, danish, muffins, or croissants	☐	☐	☐	☐	☐	☐	☐	☐	☐
Desserts and Snacks	Chocolate candy or candy bars	☐	☐	☐	☐	☐	☐	☐	☐	☐
Desserts and Snacks	Peanuts or other nuts	☐	☐	☐	☐	☐	☐	☐	☐	☐
Desserts and Snacks	Potato chips, corn chips, fried pork skins, or cheese curls	☐	☐	☐	☐	☐	☐	☐	☐	☐
Desserts and Snacks	Crackers or pretzels (include cheese and peanut butter crackers)	☐	☐	☐	☐	☐	☐	☐	☐	☐
Desserts and Snacks	Popcorn	☐	☐	☐	☐	☐	☐	☐	☐	☐
Beverages	Carbonated regular soft drinks (such as Coke, Sprite, etc.)	☐	☐	☐	☐	☐	☐	☐	☐	☐
Beverages	Diet Coke, diet sodas, or other diet drinks	☐	☐	☐	☐	☐	☐	☐	☐	☐
Beverages	Regular coffee (brewed or instant)	☐	☐	☐	☐	☐	☐	☐	☐	☐
Beverages	Decaffeinated coffee (brewed or instant)	☐	☐	☐	☐	☐	☐	☐	☐	☐
Beverages	Tea (hot or iced, but not herbal)	☐	☐	☐	☐	☐	☐	☐	☐	☐
Seasonings & Flavorings	Gravy added to potatoes, meat, or biscuits	☐	☐	☐	☐	☐	☐	☐	☐	☐
Seasonings & Flavorings	Garlic	☐	☐	☐	☐	☐	☐	☐	☐	☐
Seasonings & Flavorings	Salt added to food at the table	☐	☐	☐	☐	☐	☐	☐	☐	☐
Seasonings & Flavorings	Sugar in coffee, tea, or on cereal	☐	☐	☐	☐	☐	☐	☐	☐	☐

SAMPLE

82. **How long has it been since you last had a drink containing any kind of alcohol (this includes beer, wine and liquor)?**

- ☐ Never drank any alcohol in lifetime → **Go to question 86.**
- ☐ Within the last 24 hours
- ☐ One to 6 days ago
- ☐ One to 4 weeks ago
- ☐ One to 11 months ago
- ☐ One year or more ago → **Go to question 84.**

83. **Over the past year, how often did you drink:**

Alcoholic Beverages	Never	Rarely	1/month	2–3/month	1/week	2–3/week	4–6/week	1/day	2+/day		
Light beer	☐	☐	☐	☐	☐	☐	☐	☐	☐	When you drank beer, how many beers did you have on a typical occasion?	☐☐
Regular beer, ale, malt liquor, or stout	☐	☐	☐	☐	☐	☐	☐	☐	☐		
White wine or white wine coolers	☐	☐	☐	☐	☐	☐	☐	☐	☐	When you drank white wine or white wine coolers, how many glasses did you have on a typical occasion?	☐☐
Red wine or red wine coolers	☐	☐	☐	☐	☐	☐	☐	☐	☐	When you drank red wine or red wine coolers, how many glasses did you have on a typical occasion?	☐☐
Liquor or mixed drinks	☐	☐	☐	☐	☐	☐	☐	☐	☐	When you drank liquor or mixed drinks, how many drinks did you have on a typical occasion?	☐☐

84. **Thinking about the period in your life WHEN YOU DRANK ALCOHOL THE MOST, about how often did you drink then (this includes beer, wine and liquor)?**

- ☐ Every day
- ☐ Nearly every day
- ☐ 3-4 times a week
- ☐ 2 times a week
- ☐ once a week
- ☐ 2-3 times a month
- ☐ once a month
- ☐ 7-11 times a year
- ☐ 3-6 times a year
- ☐ 1-2 times a year or less

85. **Thinking about that period in your life WHEN YOU DRANK ALCOHOL THE MOST, about how many drinks did you have on a typical occasion, counting all types of alcohol (this includes beer, wine and liquor)?**

☐☐ drinks

SAMPLE

86. Please answer the following questions about your usual eating habits over the past year.

Eating Habits

	Never	Rarely	1/month	2–3/month	1/week	2–3/week	4–6/week	1/day	2+/day
How often do you try to restrict your food intake in order to lose weight or to keep from gaining weight?	☐	☐	☐	☐	☐	☐	☐	☐	☐
How often do you overeat, that is eating until you feel stuffed or too full?	☐	☐	☐	☐	☐	☐	☐	☐	☐
How often do you eat unplanned snacks? That is, how often do you find yourself snacking on food then thinking, "I wish I had not eaten that"?	☐	☐	☐	☐	☐	☐	☐	☐	☐
How often do you eat as a way to cope with negative feelings like anger, unhappiness, stress, or depression?	☐	☐	☐	☐	☐	☐	☐	☐	☐
How often do you eat at restaurants including fast food restaurants?	☐	☐	☐	☐	☐	☐	☐	☐	☐

87. Over the past year:

Eating Habits

When you ate greens or vegetables, how often were they cooked with:

	Most of the time	Some of the time	Never
Butter (real butter, not margarine)	☐	☐	☐
Margarine	☐	☐	☐
Lard, salt pork, or bacon fat	☐	☐	☐
Oil	☐	☐	☐

When you ate meat, how often did you trim off the fat?

- ☐ Most of the time
- ☐ Some of the time
- ☐ Never
- ☐ I don't eat meat

When you ate chicken, how often did you eat the skin?

- ☐ Most of the time
- ☐ Some of the time
- ☐ Never
- ☐ I don't eat chicken

When you ate margarine, what kind of margarine did you usually use?

- ☐ Regular Calorie Stick
- ☐ Regular Calorie Tub
- ☐ Low Calorie
- ☐ I don't use Margarine

VITAMINS AND SUPPLEMENTS

88. During the past year, have you taken any vitamin, mineral, herbal or other nutritional supplements regularly, at least once a month?

- ☐ Yes
- ☐ No → **Go to question 90.**

89. In the past year, how often have you taken:

Vitamins and Supplements

	Never	Rarely	1/month	2–3/month	1/week	2–3/week	4–6/week	1/day	2+/day
Multiple vitamins (such as, One-a-day, Centrum, Thera type Stress Tabs, or B-complex type)	☐	☐	☐	☐	☐	☐	☐	☐	☐
The following items refer to individual supplements only and not supplements that are part of a multi-vitamin.									
Vitamin A	☐	☐	☐	☐	☐	☐	☐	☐	☐
Vitamin C	☐	☐	☐	☐	☐	☐	☐	☐	☐
Vitamin E	☐	☐	☐	☐	☐	☐	☐	☐	☐
Folic Acid or Folate	☐	☐	☐	☐	☐	☐	☐	☐	☐
Calcium	☐	☐	☐	☐	☐	☐	☐	☐	☐
Zinc	☐	☐	☐	☐	☐	☐	☐	☐	☐
Gingko	☐	☐	☐	☐	☐	☐	☐	☐	☐
Ginseng	☐	☐	☐	☐	☐	☐	☐	☐	☐
St. John's Wort	☐	☐	☐	☐	☐	☐	☐	☐	☐
Garlic pills	☐	☐	☐	☐	☐	☐	☐	☐	☐

SAMPLE

90. How frequently do you grill or barbeque foods over charcoal, or on a gas or electric grill?

- ☐ Never
- ☐ Rarely
- ☐ Once per month
- ☐ 2-3 times per month
- ☐ Once per week
- ☐ 2-3 times per week
- ☐ 4-6 times per week
- ☐ Once per day
- ☐ 2 or more times per day

91. When you eat the following foods, are they usually cooked so that the INSIDE COLOR is:

	Brown	Pink	Red	I don't eat this food
hamburger or cheeseburger	☐	☐	☐	☐
Other red meat, like steak or roast beef	☐	☐	☐	☐

92. When you eat red meat like hamburgers, steak or roast beef, is it usually cooked so that the OUTSIDE COLOR is:

- ☐ Light brown
- ☐ Medium brown
- ☐ Dark brown
- ☐ Black
- ☐ I don't eat red meat

ADDITIONAL CONTACT INFORMATION

93. Can you please provide us with the name and telephone numbers of two close friends or family members (not living with you) who would know how to contact you if you moved:

Name of friend or family member: ______________________

Telephone number of friend or family member: (___) ___ - ____

Name of friend or family member: ______________________

Telephone number of friend or family member: (___) ___ - ____

94. Can you please provide us with your Social Security number:

___ - __ - ____

Collecting your social security number allows us to check health and death registries (like the National Death Index and state cancer registries) in the future to obtain information about which study participants may have passed away or been diagnosed with cancer. This type of tracing is important to the study. We will keep your social security number confidential.

Thank you for completing this questionnaire!

Please make sure that you have fully completed the study consent form in this booklet, and mail the entire booklet back to us in the enclosed postage-paid envelope.

EliteView™ forms by Pearson NCS MM233228-2 321 Printed in U.S.A.

SAMPLE

PLEASE DO NOT WRITE IN THIS AREA
